# Prosthetic Subclavian-Aortic Bypass as a Safe Surgical Technique for the Coarctation of the Aorta in Adults

**DOI:** 10.3889/oamjms.2016.006

**Published:** 2015-12-24

**Authors:** Ali Refatllari, Ermal Likaj, Selman Dumani

**Affiliations:** *Department of Cardiovascular Surgery, University Hospital Centre “Mother Theresa”, Rruga e Dibres, N. 370, Tirana, Albania*

**Keywords:** coarctation, adults, bypass graft, hypertension, technique

## Abstract

**BACKGROUND:**

Coarctation represents 5-8% of congenital heart disease. Residual hypertension remains the main problem after late correction. Surgical treatment in the adult remains a challenge for the surgeon. Our prefered method used in this category is the Subclavian-aortic bypass.

**MATERIAL AND METHODS:**

We have reviewed our registry for the period of 12 years (1998- 2010) and we found a group of 18 adult patients being operated for coarctation of the aorta. The mean age of this group of patients was 24.7 ± 8.43 years (range 16-42 years). 13 were males and 5 females.

**RESULTS:**

Sugical technique: Most of the patients (13 pts, 72%) which were obviously treated with subclavian-aortic bypass with a Dacron prostheses. Mean preoperative and postoperative pressure gradients measured by echocardiography were 77.7 ± 20.16 mmHg and 22.3 ± 9.14 mmHg respectively. No mortality was observed in this series of patients. Chylothorax was the only complication observed in one patient in the early postoperative period.

**CONCLUSION:**

Coarctation of the aorta in adults is treated with optimal early results at our surgical centre. Subclavian-aortic bypass grafting requires less aortic dissection, can be performed with a partially occluding clamp, and does not compromise the spinal cord vascularization.

## Introduction

Aortic coarctation is a congenital luminal narrowing (usually around the origin of the left subclavian artery), which obstructs blood flow. Coarctation represents 5-8% of congenital heart disease [1]. Since the first successful repair of aortic coarctation [2], treatment is now well established, but residual hypertension remains the main problem after late correction [3].

However, despite a 60-year experience, surgical repair of coarctation remains a technical challenge for the surgeon in the subset of older patients with atypical anatomic forms of coarctation: long coarctation, aortic wall calcifications, extensive or minimal collateral circulation, and multiple previous operations. These patients are exposed to major operative risks with use of the various surgical anatomic techniques involving direct exposure of the diseased aorta: end-to-end anastomosis, subclavian flap aortoplasty, subclavian displacement, prosthetic patch aortoplasty, or prosthetic patch interposition grafting. Specific risks are linked either to the dissection of the aortic and periaortic structures or to spinal cord ischemia associated with total aortic cross-clamping. To minimize these drawbacks in these patients, our prefered method used in this category is a bypass graft (subclavian artery-descending aorta).

The purpose of this study was to review our 12-year experience with this proce-dure and report the early results.

## Material and Methods

We have reviewed our registry for the period of 12 years (1998-2010) and we found a group of 18 adult patients being operated for coarctation of the aorta. The mean age of this group of patients was 24.7 ± 8.43 years (range 16-42 years), 13 were males and 5 females. In three patients we had an association with bicuspid aortic valve and in one of them a dilated ascending aorta which was replaced in the same procedure via sternotomy. All patients presented with arterial hypertension and used two or more antihypertensive drugs.

**Figure 1:**
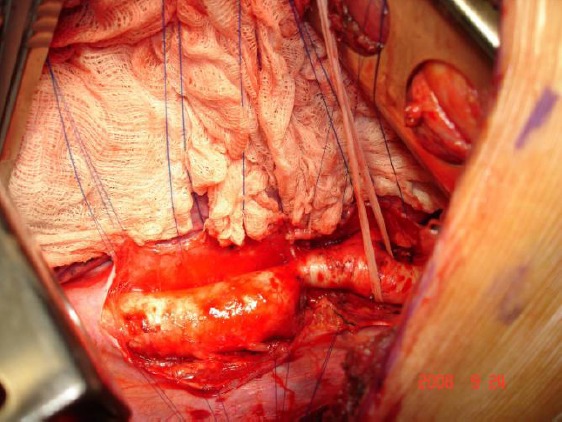
*Surgical view of coarctation of the aorta*.

## Results

### Operative technique

Most of the patients (13 pts, 72%) were obviously treated with subclavian-aortic bypass with a Dacron prostheses. A left posterolateral thoracotomy was used in these patients. The dissection was limited to the area of the anastomoses. Proximal implantation was done on the left subclavian artery. Distal implantation was performed on the descending thoracic aorta in all instances. Both proximal and distal end-to-side anastomoses were performed under partial aortic cross-clamping using continuous 5-0 polypropylene suture.

In 3 patients resection and primary anastomosis was used. The surgical option for one patient was synthetic patch aortoplasty and for another patient resection and graft interposition.

**Table 1:** Patients according to the applied surgical technique

Type of operation	Number of patients
Subclavian-aortic bypass with dacron prostheses	13 pts
Resection and end to end anastomosis	3 pts
Synthetic patch aortoplasty	1 pts
Resection and graft interposition.	1 pts

No mortality was observed, and the postoperative period was generally uneventful this series of patients.

**Figure 2:**
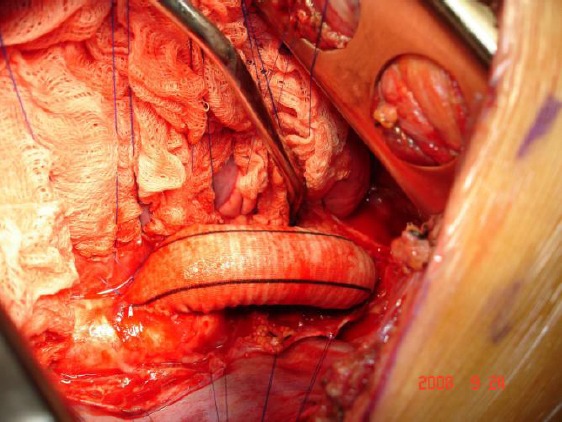
*Prosthetic subclavian aortic bypass grafting*.

Mean preoperative and postoperative pressure gradients measured by echocardiography were 77.7 ± 20.16 mmHg and 22.3 ± 9.14 mmHg respectively.

**Figure 3:**
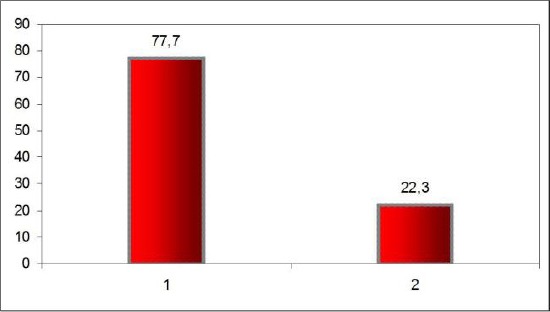
*Reduction of gradients measured by echocardiography postoperatively*.

Chylothorax was the only complication observed in one patient in the early postoperative period. The patient was treated successfully in a conservative regimen. Early after the correction 12 patients (66%) were normotensive and the rest of them (6 patients, 34%) used only one drug to control the hypertension.

## Discussion

Unrepaired coarctation of the aorta results in high morbidity and mortality from hypertension and associated problems, including myocardial infarction, heart failure, intracranial hemorrhage, aortic rupture, and infective endocarditis. Without correction, most patients die before the age of 50 years [3]. Usually adult patients present with hypertension which is the main complication associated with the pathology at this age. All of our patients were diagnosed based on a work-up for hypertension. Several authors confirm that surgical repair of aortic coarctation in patients older than 20 years of age reduces systolic hypertension [4, 5].

On the basis of the worldwide experience with coarctation of aorta in adults, repair is recommended even at advanced ages. In a series, reported by Wells et al patients up to the age of 60 years appear to have benefited from the operation as indicated by improved control of systolic hypertension [5]. In our group of patients early after the repair 66% of the patients were normotensive at rest without medication. Because the risk of operation is extremely low, operation is recommended even for patients with mild preoperative hypertension [5].

Different techniques are available for the correction of this anomaly in adults. Resection and end-to-end anastomosis, which is the best choice in the pediatric population cannot always by applied in adults, and furthermore is linked with and increased risk for complications. Compared with the aorta of infants and children, the aorta of the adult patient with coarctation is relatively immobile, and there are frequently large collaterals immediately adjacent to the narrow segment. This makes bypass grafting from the left subclavian or distal aortic arch to the descending aorta, which can be performed with a partially occluding clamp, an attractive option. This is the likely explanation for its frequent use at our institution. These techniques are simple, safe, and feasible [6].

On the other hand, Campbell et al have concluded that patch aortoplasty repair of aortic coarctation should be abandoned in adults, because of the high incidence of aneurysm formation after the procedure [3].

Coarctation of the aorta in adults is treated with optimal early results at our surgical centre. Subclavian-aortic bypass grafting requires less aortic dissection, can be performed with a partially occluding clamp, and does not compromise the spinal cord vascularization. We conclude that this is a safe choice for the treatment of aortic coarctation in the adult population where other techniques are more hazardous.
